# The Relationship between Inflammatory Cytokines and Coagulopathy in Patients with COVID-19

**DOI:** 10.3390/jcm10092020

**Published:** 2021-05-09

**Authors:** Fariba Rad, Ali Dabbagh, Akbar Dorgalaleh, Arijit Biswas

**Affiliations:** 1 Cellular and Molecular Research Center, Yasuj University of Medical Sciences, Yasuj 7591994799, Iran; 2 Anesthesia Research Center, Shahid Beheshti University of Medical Sciences, Tehran 1998734383, Iran; alidabbagh@yahoo.com; 3 Department of Hematology and Blood Transfusion, School of Allied Medicine, Iran University of Medical Sciences, Tehran 1449614535, Iran; dorgalaleha@gmail.com; 4 Institute of Experimental Hematology and Transfusion Medicine, University of Bonn, 53127 Bonn, Germany; arijit.biswas@ukb.uni-bonn.de

**Keywords:** COVID-19, SARS-CoV-2, cytokine storms, inflammation, coagulopathy

## Abstract

Coronavirus disease 2019 (COVID-19), with a broad range of clinical and laboratory findings, is currently the most prevalent medical challenge worldwide. In this disease, hypercoagulability and hyperinflammation, two common features, are accompanied by a higher rate of morbidity and mortality. We assessed the association between baseline inflammatory cytokine levels and coagulopathy and disease outcome in COVID-19. One hundred and thirty-seven consecutive patients hospitalized with COVID-19 were selected for the study. Baseline interleukin-1 (IL-1), IL-6, and tumor necrosis factor alpha (TNF-α) level were measured at time of admission. At the same time, baseline coagulation parameters were also assessed during the patient’s hospitalization. Clinical findings, including development of thrombosis and clinical outcome, were recorded prospectively. Out of 136 patients, 87 (~64%) had increased cytokine levels (one or more cytokines) or abnormal coagulation parameters. Among them, 58 (~67%) had only increased inflammatory cytokines, 12 (~14%) had only coagulation abnormalities, and 17 (19.5%) had concomitant abnormalities in both systems. It seems that a high level of inflammatory cytokines at admission points to an increased risk of developing coagulopathy, thrombotic events, even death, over the course of COVID-19. Early measurement of these cytokines, and timely co-administration of anti-inflammatories with anticoagulants could decrease thrombotic events and related fatal consequences.

## 1. Introduction

Severe acute respiratory syndrome-2 (SARS-CoV-2) is a new medical challenge that, to date, has affected 55 million people and caused more than one million deaths around the world [1]. Those affected have various clinical presentations, most commonly cough, fever, shortness of breath, fatigue, and myalgia. Less commonly, diarrhea, anorexia, diminished smell and taste, and headache are reported. Gastrointestinal (GI) bleeding, hiccups, and central nervous system (CNS) bleeding occur rarely [2,3]. Patients with underlying risk factors such as elevated blood pressure, obesity, cardiovascular diseases, diabetes, renal failure, etc., may experience a more severe course of coronavirus disease 2019 (COVID-19) [4]. Coagulopathy is a common and significant feature of COVID-19, which is more severe and profound in patients admitted to an intensive care unit (ICU). Prolonged prothrombin time (PT), activated partial thromboplastin time (APTT), increased D-dimer, fibrin degradation products (FDPs), and fibrinogen are common findings [5,6]. Decreased platelet count is another common finding that is associated with a higher rate of morbidity and mortality [7]. Venous thromboembolism (VTE), which has been reported in approximately 17% of patients, is a fatal complication and the direct cause of death in about one-third of those affected [8]. Viral infection can change the coagulation system in contradictory ways, increasing coagulation activity (hypercoagulation), or attenuating coagulation (hypocoagulation). Since hypercoagulability is one of the most prominent features of COVID-19, most probably due to increased coagulation factor levels and platelet hyper-activation, as shown by several studies, this might be a part of an acute phase reaction to the virus [9,10,11]. Increased coagulation factor levels observed in patients with COVID-19 most probably are part of a hyperinflammation reaction. In addition to acute phase reactants—coagulation factors like fibrinogen and factor VIII—increased factor V level has been observed, without a direct correlation between it and the acute phase reactants [12]. Elevated generation of tissue factor and reduced activity of tissue factor pathway inhibitor (TFPI) directly through the proinflammatory cytokines lead to increased levels of tissue factor [13,14]. This phenomenon has been speculated to be the case in pro-thrombotic phenotype observed in COVID-19 patients [14]. Inflammatory factors also reduce the level of thromboregulatory proteins, which directly inhibit the synthesis, activation, and function of protein C, while increasing the consumption of it, leading to immunothrombosis [14]. Hypo-fibrinolytic changes characterized by induced formation of plasminogen activator inhibitor-1 (PAI-1) by inflammatory factors, and reduced lysis of PAI-1 due to inhibition of protein C observed among reported pathophysiological mechanisms responsible for COVID-19 associated coagulopathy [14,15]. In this study we assessed the association between baseline levels of inflammatory cytokines and the coagulation system, thrombotic complication, and disease outcome in patients affected by COVID-19.

## 2. Experimental Section

### 2.1. Study Population

The study, approved by the Ethical Committee of Yasuj University of Medical Sciences, included 136 consecutive patients hospitalized with COVID-19, diagnosed with a positive SARS-CoV-2 polymerase chain reaction (PCR). Patients with a known coagulopathy or immunodeficiency were excluded. Our hypothesis was that when patients had a higher level of inflammatory cytokines, they more probably would experience coagulopathy and related consequences. Thus, interleukin-1 (IL-1), IL-6, and tumor necrosis factor alpha (TNF-α) levels were measured at admission. Baseline coagulation profiles were determined on admission, prior to starting treatment. Coagulation test results, with clinical presentation and disease outcome were also recorded prospectively.

### 2.2. Laboratory Analysis

Inflammatory cytokine levels, IL-1, IL-6, and TNF-α, were measured by enzyme-linked immunosorbent assay (ELISA) at admission. At the same time, coagulation tests, prothrombin time (PT), activated partial thromboplastin time (APTT), fibrinogen, D-dimer, fibrin(ogen) degradation products (FDP), and factor XII were measured for a baseline coagulation profile. Fibrinogen was done by one-stage PT- and PTT-based assays (STA Compact automatic coagulometer, Stago, Paris, France). Factor XII activity was measured by APTT-based assay (HemosIL, MA, USA, ACLTOP 700 instrument). FDP was measured using a semi-quantitative latex agglutination assay, and D-dimer by a quantitative latex assay (STA-LIA test D-DI, Diagnostica-Stago, Paris, France). Additional coagulation tests, including D-dimer, PT, and APTT, were performed at the request of patients’ physicians. All results above the upper or lower limit of the reference range were considered abnormal. Patients were divided into two groups according to their baseline cytokine levels: (1) those with normal cytokine levels, (2) those with increased cytokine levels.

### 2.3. Clinical Characteristics

At baseline, severity of COVID-19 was classified according to the guidance issued by the National Health Commission [16]. Patients’ presentations were collected during hospitalization (median: 27 days (7–58)). Venous thromboembolism (VTE) events, deep vein thrombosis (DVT) and pulmonary embolism (PE) were recorded during their convalescence. Diagnostic strategy for venous thromboembolism was careful screening of patients’ clinical manifestations to determine signs and symptoms indicative of VTE including airway bleeding, pulmonary derangement and acute onset of unspecified tachycardia and hypotension, followed by the use of Doppler ultrasonography (DUS) of both lower extremities with the application of CT angiography especially in high-risk ambulatory patients with negative sonography [17,18]. All mortalities during the study period were recorded, as were the underlying risk factors.

### 2.4. Statistical Analysis

Results were reported as median with the interquartile range (IQR) for quantitative variables, and percentages for categorical variables. Chi squared (χ²) test and the Mann–Whitney U-test were used to examine differences between two groups, as appropriate. Logistic regression analysis was used for multivariate analysis. Statistical significance was defined as *p*-value of 0.05 or lesser. The Kolmogorov–Smirnov test was used to evaluate the distribution of normality. All statistical analyses were performed by SPSS version 16 software.

## 3. Results

### 3.1. Characteristics of the Study Population

One hundred and thirty-seven patients with COVID-19 were hospitalized during the study period, but one was excluded due to his underlying coagulopathy. Thus, 86 males (~63%), and 50 females (~37%) were included in the study. The patients’ median age was 56 years (range from 21–64 years), 61 for males, and 54 for females (*p* < 0.05). Twenty-two patients (16.3%) had underlying disorders, including cardiovascular disorders (*n*: 9, 41%), diabetes (*n*: 5, 22.7%), hypothyroidism (*n*: 3, 13.6%), renal failure (*n*: 1, 4.5%), and hepatic failure (*n*: 1, 4.5%). Patients were hospitalized for 7–58 days (median: 27 days), 53 patients (~39%) were admitted to ICU, 31 (~23%) experienced DVT/PE, seven died (5.1%). Thirty-eight (~72%) patients admitted to ICU were male, 15 (~28%) were female (*p* = 0.02). Patients were divided into two groups according to their baseline cytokine levels; group one with increased baseline cytokine levels and group two with normal levels (Table 1).

### 3.2. Laboratory Findings

**Figure 1:**
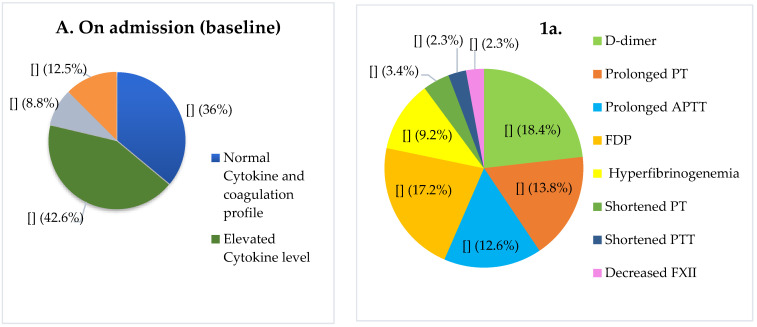

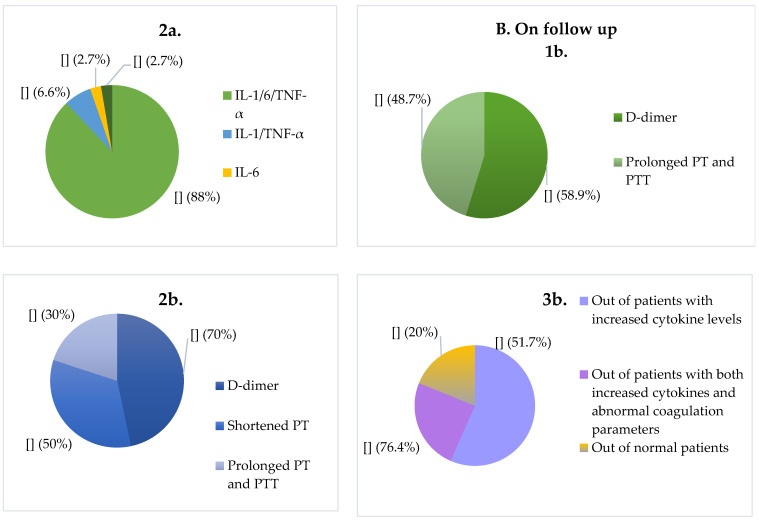
On admission (baseline) (**A**) and follow up characteristics (**B**) of patients with COVID-19. Patients’ distribution in terms of inflammatory cytokines levels and coagulation parameters. Prevalence of abnormal coagulation test results among those with abnormal parameters (**1a**) (*n* = 87); the status of inflammatory cytokines among patients with increased cytokine levels (**2a**). Developed coagulopathies in thirty nine of 58 patients with only increased cytokines (67.2%) (**1b**); in ten of 49 patients with normal bassline cytokines and coagulation parameters (**2b**). Out of 136 patients 53 (~39%) were admitted to ICU, of whom 30 patients had baseline increased cytokine levels, 13 patients had concomitant baseline increased cytokines and abnormal coagulation parameters, and 10 patients had normal cytokines and coagulation parameters (**3b**). PT: Prothrombin time, APTT: Activated Partial Thromboplastin Time, IL-1: Interleukin 1, FDP: fibrin(ogen) degradation products, TNF-α: tumor necrosis factor alpha.

Out of 136 patients, 58 (42.6%) had only increased levels of inflammatory cytokine (one or more cytokines), 12 (8.8%) had only abnormal coagulation parameters, and 17 (12.5%) had concomitant abnormalities in both systems (Figure 1). The most common abnormal coagulation tests among symptomatic patients (*n* = 87) were increased D-dimer (*n*: 16, 18.4%), prolonged PT (*n*: 12, 13.8%) and APTT (*n*: 11, 12.6%), FDP (*n*: 15, 17.2%), and increased fibrinogen (*n*: 8, 9.2%). Shortened PT (*n*: 3, 3.4%) and APTT (*n*: 2, 2.3%) were other abnormal findings. Factor XII level was decreased in 2 patients (2.3%), both with increased inflammatory cytokines. There is no statistically significant difference between baseline median of PT, APTT, D-dimer, FDP in patients with increased and normal cytokine levels (*p* > 0.05). Concomitant increase of interleukin-1 (IL-1), IL-6, and TNF-α was observed in 66 (88%) patients, increased TNF-α and IL-1 was observed in 5 (6.6%), increased IL-6 in 2 (2.7%), and TNF-α in 2 (2.7%). Thirty nine of 58 patients with only increased cytokines (67.2%) developed coagulopathies; among patients with normal cytokines and a normal coagulation profile (*n*: 49), only 10 (20.4%) developed coagulopathy during hospitalization (*p* < 0.001). The abnormal coagulation resolved in three patients (25%) with baseline abnormal coagulation tests and normal cytokines, but this rate was 11.6% (*n*: 2) among patients with baseline concomitant abnormalities in both systems (*p* > 0.05). Among patients with only increased cytokines, the most common abnormal results on follow up were increased D-dimer (*n*: 23, 58.9%), and prolonged PT and APTT (*n*: 19, 48.7%). Among patients with normal baseline cytokines, increased D-dimer (*n*: 7, 70%), shortened PT (*n*: 5, 50%), and prolonged PT and APTT (*n*: 3, 30%) were abnormal coagulation parameters during hospitalization. The median of PT, APTT (*p* < 0.01) and D-dimer (*p* < 0.001) was significantly different between the two groups (Table 2). More than 80% (*n*: 43) of patients admitted to ICU had baseline increased cytokine levels (*n*: 30, 51.7%) or had concomitant baseline increased cytokines and abnormal coagulation parameters (*n*: 13, 76.4%), while only ~20% (*n*: 10) of patients with normal cytokines and coagulation parameters were admitted to ICU (*p* < 0.001).

### 3.3. Clinical Outcome

All patients experienced moderate to severe COVID-19 disease. Seven (41.2%) patients with concomitant abnormalities in both systems, 19 (32.8%) with only increased baseline cytokines and 5 (14.2%) with normal cytokine levels, developed deep vein thrombosis (DVT)/pulmonary embolism (PE) (*p* < 0.01). Six patients with baseline increased cytokines died, while the seventh patient who died had abnormal coagulation parameters without increased cytokines (*p* < 0.01). Logistic regression analysis revealed that during hospitalization coagulation abnormality in the PT and PTT results was more related to the cytokine levels.

Regression analysis also showed IL-1 level has the highest correlation with the VTE outcome compared to other inflammatory cytokines (Table 3).

## 4. Discussion

Cytokine storm and coagulopathy are two main complications of COVID-19, and are more profound and serious in those with a more severe form of the disease [2,5]. Upon viral infection, both innate and adaptive immune systems are involved. When the innate system, the body’s first barrier against external agents, cannot eliminate the virus, foreign agents’ recognition sites (PAMP) provoke the immune system, causing the release of more and more cytokines, which leads to the phenomenon termed “cytokine storm” [19,20]. This storm, commonly observed among patients with COVID-19, occurs most frequently among the severely ill, particularly those who suffer lethal consequences. There is a direct correlation between the immune and coagulation systems; thus, immunological and coagulation disturbances are common in COVID-19. Both innate and adaptive immune systems are affected in SARS-CoV-2 infection [21]. Since inflammation has a direct effect on coagulation, we decided to assess the relationship between the two systems. We found that those with a high baseline level of inflammatory cytokines may experience more profound coagulation disturbance, thrombotic risk and fatal consequences. IL-6 concentration has been shown to be higher in patients with severe COVID-19. Moreover, IL-6 levels in non-survivors were 1.7x higher than in survivors [21,22]. In our study, we found that the rate of mortality was higher in patients with elevated baseline cytokines. IL-6 and D-dimer testing provide an early sensitive and specific predictor of a severe course of COVID-19 [21,22]. Those of our patients whose baseline levels of inflammatory cytokines were elevated more commonly developed coagulopathy than those with normal baseline cytokines (67% vs. 16.3%). Shortened PT and APTT have been reported as a common finding; our study found that shortened PT and APTT commonly indicated baseline higher inflammatory cytokines. This might be due to increased levels of acute phase reactant coagulation factors. An inflammatory reaction could be accompanied by increased levels of coagulation factors, as noted by a recent study, which found a clear increase in coagulation factor V [12]. Similar studies found higher levels of coagulation factors in patients with SARS-CoV-2 infection, potentially making the patients more prone to thrombotic events. Early testing of inflammatory markers, and a coagulation profile, could be used to identify patients with more severe infection. Thus, timely intervention could decrease the disease-related morbidity and mortality. It has been suggested that direct targeting of inflammatory cytokine receptors such as IL-6, by a monoclonal antibody such as tocilizumab may be useful in reducing mortality [23]. Recently, Magro and colleagues postulated that the complement system might be involved in pathophysiological mechanisms responsible for respiratory microvasculature injuries and subsequent thrombus formation in severely ill COVID-19 patients [24]. This report, by highlighting the evidence regarding complement-mediated endothelial cell injury and neutrophil attraction accentuated the inflammatory aspects of complement pathways activation and subsequent coagulopathy. Membrane attack complex-mediated alveolar subendothelial exposure, secretion of proinflammatory cytokines, the neutrophil chemoattractant properties of complement, and also C5a receptor/tissue factor cross-talk mediated by neutrophils are reported to be involved in SARS-CoV-2 induced thromboinflammation. It has been hypothesized that intervention with inhibitors of the complement pathways activation may be relevant to severe COVID-19 infection, especially when supported by murine SARS-CoV infected models [24,25]. However, direct invasion of the virus, the fundamental objective of treatment, is not available at this time. In fact, identification of risk factors is the main key to controlling COVID-19 and reducing related fatal consequences. Hypercoagulability is among the leading causes of death. Since there is a correlation between inflammatory cytokines and hypercoagulability, early diagnosis of raised cytokine levels is desirable. Anticoagulation is recommended by the International Society of Thrombosis and Hemostasis (ISTH) interim guidelines for all patients hospitalized with COVID-19 [26]. However, currently, no anti-inflammatory drugs are recommended. Low molecular weight heparin (LMWH), the anticoagulant of choice for most patients, has both anticoagulant and anti-inflammatory effects [27,28]; it shows promise for patients with COVID-19 although the effect of this drug in controlling hyperinflammation is not clear. Moreover, this effect is not strong, thus, its role in controlling hyperinflammation in COVID-19 is far from expected.

However, patients with hyperinflammation might benefit from concomitant administration of anticoagulants and anti-inflammatories. It is strongly recommended that all hospitalized patients have their cytokine levels monitored over the course of their illness to enable timely intervention to any emerging hyperinflammatory response.

## Figures and Tables

**Table 1:** Baseline characteristics of patients with COVID-19.

		Normal Cytokine Levels	Increased Cytokine Levels	Total	*p* Value
Number		61	75	136	*p* > 0.05
Age (year) (range)		58 (21–59)	55 (23–64)	56 (21–64)	*p* > 0.05
Gender (male/female)		43/18	45/30	86/50	*p* > 0.05
Abnormal coagulation tests: *n* (%)	D-dimer	5 (31.2%)	11 (68.8%)	16	NA
	PT	6 (40%)	9 (60%)	15	
	APTT	6 (46.2%)	7 (53.8%)	13	
	FDP	6 (40%)	9 (60%)	15	
	Fibrinogen	2 (25%)	6 (75%)	8	
	FXII	0	2 (100%)	2	
DVT/PE		5	26	31	*p* < 0.001
Underlying disorders	Diabetes	3	2	22	NA
	Renal failure	0	1		
	Hepatic failure	0	1		
	Cancer	2	1		
	Hypothyroidism	1	2		
	Cardiovascular disorders	6	3		
Death		1	6	7	*p* < 0.001

DVT: Deep vein thrombosis, PE: Pulmonary embolism, PT: Prothrombin time, APTT; Activated partial thromboplastin time, Fib: Fibrinogen, FDP; Fibrin(ogen) degradation products, FXII: Factor XII.

**Table 2:** Baseline and follow-up coagulation parameters of hospitalized patients with COVID-19.

	Normal Cytokines Levels	Increased Cytokines Levels	Total	Reference Range	*p* Value	Multivariate OR (95% CI)	*p* Value
PT (median) (s)	11.8	12.6	12.4	11.2–13.8	*p* > 0.05	NA	NA
APTT (median) (s)	29.4	27.5	28.2	21–34	*p* > 0.05		
D-dimer (median) (ng/mL) (FEU)	542	625	595	500	*p* > 0.05		
FDP (median) (μg/mL)	8.3	9.2	8.7	5	*p* > 0.05		
Fibrinogen (median) (mg/dL)	345	415	384	200–400	*p* > 0.05		
Factor XII activity (median) (IU/dL)	95	87	91	60–150	*p* > 0.05		
Coagulation parameters during hospitalization							
PT (median) (s)	14.8	19.5	17.2	11.7–14.3	*p* < 0.01	1.275 (1.023–1.590)	0.031
APTT (median) (s)	39	58.5	43.5	22–36	*p* < 0.01	1.074 (1.027–1.124)	0.002
D-dimer (median) (ng/mL) (FEU)	648	1245	826	500	*p* < 0.001	-	-

PT: Prothrombin time, APTT: Activated partial thromboplastin time, FDP: fibrin(ogen) degradation products.

**Table 3:** Univariate analysis and multivariate logistic regression for variables related to DVT/PE.

Dependent Variable	Independent Variable	Univariate *p*	Multivariate OR (95% CI)	*p* Value
DVT/PE	IL-1	0.000 *	6.778 (2.409–19.072)	0.000
	IL-6	0.000 *	-	-
	TNF-α	0.000 *	-	-

* The variables entered into the logistic regression. PT: Prothrombin time, APTT: Activated Partial Thromboplastin Time, IL-1: Interleukin 1, FDP: fibrin(ogen) degradation products, TNF-α: tumor necrosis factor alpha.

## Data Availability

The data presented in this study are available on request from the corresponding author.
